# Macular corneal dystrophy related to novel mutations of *CHST6* in a Chinese family and clinical observation after penetrating keratoplasty

**DOI:** 10.1186/s12920-021-01095-7

**Published:** 2021-10-13

**Authors:** Dewei Li, Le Tian, Xiaochuan Wang, Min Chen

**Affiliations:** 1 Qingdao Eye Hospital of Shandong First Medical University, Qingdao, China; 2 State Key Laboratory Cultivation Base, Shandong Provincial Key Laboratory of Ophthalmology, Shandong Eye Institute, Shandong First Medical University and Shandong Academy of Medical Sciences, 5 Yan’erdao Road, Qingdao, 266071 China

**Keywords:** *CHST6*, Macular corneal dystrophy, Penetrating keratoplasty, Clinical phenotype, Gene mutation

## Abstract

**Background:**

Macular corneal dystrophy (MCD) is a rare corneal stromal dystrophy with bilateral progressive vision loss. The pathogenic gene of MCD is carbohydrate sulfotransferase 6 (*CHST6*). Herein, we report a novel missense mutation and a rare exon deletion mutation in the *CHST6* gene in a Chinese family with MCD.

**Methods:**

Genomic DNA was extracted from the peripheral blood, and next generation sequencing was used to analyse the gene sequence. The pathogenic mutations were identified in all affected family members. The proband successively received binocular penetrating keratoplasty (PKP), and the corneas were examined by histopathology and colloidal iron staining to prove the diagnosis. A long-term follow-up was made to observe the changes after PKP.

**Results:**

Genetic analysis demonstrated hemizygous mutations in the proband, including a novel c.520A>C (p.K174Q) missense mutation and a rarely reported exon 3 deletion mutation, which were co-segregated with the MCD phenotypes in the pedigree. The positive colloidal iron staining confirmed the diagnosis of MCD in the proband. However, the clinical phenotype and pathological manifestation of both eyes were different from each other because of complicated keratitis in the left eye. During the nine years of follow-up, visual acuity was improved significantly, and the cornea was transparent without rejection and postoperative recurrence in both eyes.

**Conclusions:**

The novel hemizygous mutations were thought to contribute to the loss of *CHST6* function, which induced typical clinical and pathological features of MCD. PKP was an effective treatment for MCD.

## Background

Corneal dystrophy typically refers to a group of inherited corneal disorders that are usually bilateral, symmetric, slowly progressive, and unrelated to environmental or systemic factors [1]. Macular corneal dystrophy (MCD, **MIM 217800**) is a rare stromal dystrophy with bilateral progressive vision loss, and its genetic characteristic is autosomal recessive inheritance. Patients with MCD often begin to experience vision loss by the age of 10 years and suffer from severe visual impairment by 20 or 30 years of age. Some patients have photophobia, foreign body sensation, eye pain, and reduced corneal sensitivity, which are caused by recurrent corneal epithelial erosion [1, 2].

The pathogenic gene of MCD is the carbohydrate sulfotransferase 6-(*CHST6*, **MIM 605294**) gene, which is located on chromosome 16q23.1 (which contains four exons), and exon 3 encodes the *CHST6* (C-GlcNAc6ST, **EC 2.8.2.-**), comprising 395 amino acids. C-GlcNAc6ST catalyses 6-hydroxy of N-acetyl glucosamine, galactose, and N-acetylgalactosamine 6-O sulphation, and is responsible for transferring the sulphate group from 3'-adenosine 5'-phosphate to keratan sulphate [3]. The *CHST6* gene mutation changes the enzyme function, resulting in keratan sulphation disorders, which lead to MCD [4]. Currently, there are more than 180 kinds of pathogenic or possibly pathogenic mutations with no difference in the distribution of exon 3 [2, 5, 6]. This means that *CHST6* has mutation diversity, which clearly distinguishes it from the hot-spot mutations of transforming growth factor beta-induced (*TGFBI*) corneal dystrophy. Our study disclosed a special *CHST6* variant type in a Chinese family with MCD, and evaluated clinical and corneal histopathological characteristics and therapeutic outcomes after penetrating keratoplasty (PKP) of the proband.

## Methods

### Patient information

In May 2012, a 50-year-old woman who complained of impaired binocular visual acuity for more than 20 years was diagnosed with binocular MCD at Qingdao Eye Hospital. The proband underwent PKP on the right eye in 2012 and PKP combined with cataract surgery on the left eye in 2019. We invited her family members to undergo clinical examination, pedigree analysis, and genetic testing. This study was approved by the Ethics Committee of Qingdao Eye Hospital (2019-15) and followed the tenets of Declaration of Helsinki. Informed consent was obtained from all participants.

### Ocular examination

Ocular examination by slit lamp microscopy (BM900, Haag-Streit AG, SWISS) was performed in the family members to find any potential eye disease. The eyes of the proband were examined during the follow-up period after PKP by counting endothelial cells (NSP-9900 II, Konan Medical Inc., JAPAN), anterior segment photography (Photo Slit Lamp BX900, Eyesuite software, Haag-Streit AG, SWISS; EOS7D Mark II camera, Canon, JAPAN), slit lamp microscopy for corneal morphology, optical coherence tomography (OCT, RTVue100-2, Optovue Inc., USA) for corneal opacity, and ultrasound biomicroscopy (SW-3200L, SUOER, China) for anterior chamber angles. Corneal samples were subjected for hematoxylin–eosin staining, colloidal iron staining, and Alcian blue staining (BX60 Fluorescence Microscope; DP72 camera; cellSens Standard 1.6 software, Olympus, Japan). Therapeutic outcomes were assessed.

### Gene sequencing analysis

Approximately 2 mL of the peripheral blood was collected from one affected family member and two unaffected family members to extract genomic DNA. Target sequencing was used to analyse the DNA sequence. A gene panel containing 111 genes associated with corneal disease was used, including the *CHST6* gene. DNA libraries were prepared according to the Illumina’s protocol, and the 111 genes were selected by a gene capture strategy, using the GenCap custom enrichment kit (MyGenostics Inc., Beijing, China).

Paired-end sequencing with 150 bp per read was performed on a NextSeq 500 sequencer (Illumina, San Diego, CA). After sequencing, the rawdata were saved as a FASTQ format, then Illumina sequencing adapters and low quality reads (< 80 bp) were filtered by cutadapt. After quality control, the clean reads were mapped to the human reference genome (hg19) using the BWA software (http://bio-bwa.sourceforge.net/). The fastq file was converted to the bam file and then the vcf file. The ANNOVAR software (http://annovar.openbioinformatics.org/en/latest/) was used to annotate single-nucleotide polymorphisms (SNPs) identified using the SOAPsnp program (http://soap.genomics.org.cn/soapsnp.html), and insertions or deletions (indels) identified using the GATK (http://www.broadinstitute.org/gsa/wiki/index) were annotated using the Exome-assistant program (http://122.228.158.106/exomeassistant). The SNPs and indels with a frequency of more than 0.02 in HapMap samples, 1000 Genome, ESP6500, ExAC_ALL, or ExAC_EAS were removed. Nonsynonymous variants were evaluated by SIFT (http://sift.bii.a-star.edu.sg/), Ployphen-2 (http://genetics.bwh.harvard.edu/pph2/), MutationTaster (http://www.mutationtaster.org/), and GERP +  + (https://www.biostars.org/p/207518/) to predict their pathogenicity. The Human Gene Mutation Database (HGMD; http://www.hgmd.cf.ac.uk/ac/index.php) was used to search for identified variant novelty. Finally, in the assessment of variant interpretations and pathogenicity, the American College of Medical Genetics and Genomics (ACMG) 2015 guidelines were used.

The candidate pathogenic mutations were identified by Sanger sequencing. A fragment spanning the location of the *CHST6* K174Q mutation was amplified by polymerase chain reaction with the primers as 5’- GGT GAT GTT ATG GAT CCA GGC-3’ (forward) and 5’- CTG TCC GAC CTC TTC CAG TG -3’ (reverse). Real-time quantitative PCR reaction was carried out using the CFX96 Touch Real-Time PCR Detection System (Bio-Rad, Hercules, CA, USA). And SYBR Green I (TaKaRa) was used as the fluorescent label. The mutation was sequenced on the ABI 3730 analyzer (Applied Biosystem). The average sequencing depth on the target regions was 254 × , and the coverage of target regions was 99.9%. Variations were further confirmed by comparing the sequence with the reference sequence of *CHST6* (**NM_021615**) using the Mutation Surveyor software. In cases of missense mutations, conservation of the involved amino acids among several sulfotransferases of human and other animals was investigated using Clustal Omega (https://www.ebi.ac.uk/Tools/msa/clustalo/).

The Copy Number Variation analysis was performed (MyGenostics Inc.), and the samples captured in the same pool was analyse with Cnvkit software. The read depth of target regions was counted, homogenized, and corrected in the sample, and then compared with the control set. Multiple samples to establish reference for error correction of the read depth were used. We chose the *TAT* and *ADAMTS18* genes which were located near the *CHST6* as the control set.

## Results

### Pedigree analysis

**Fig. 1:**
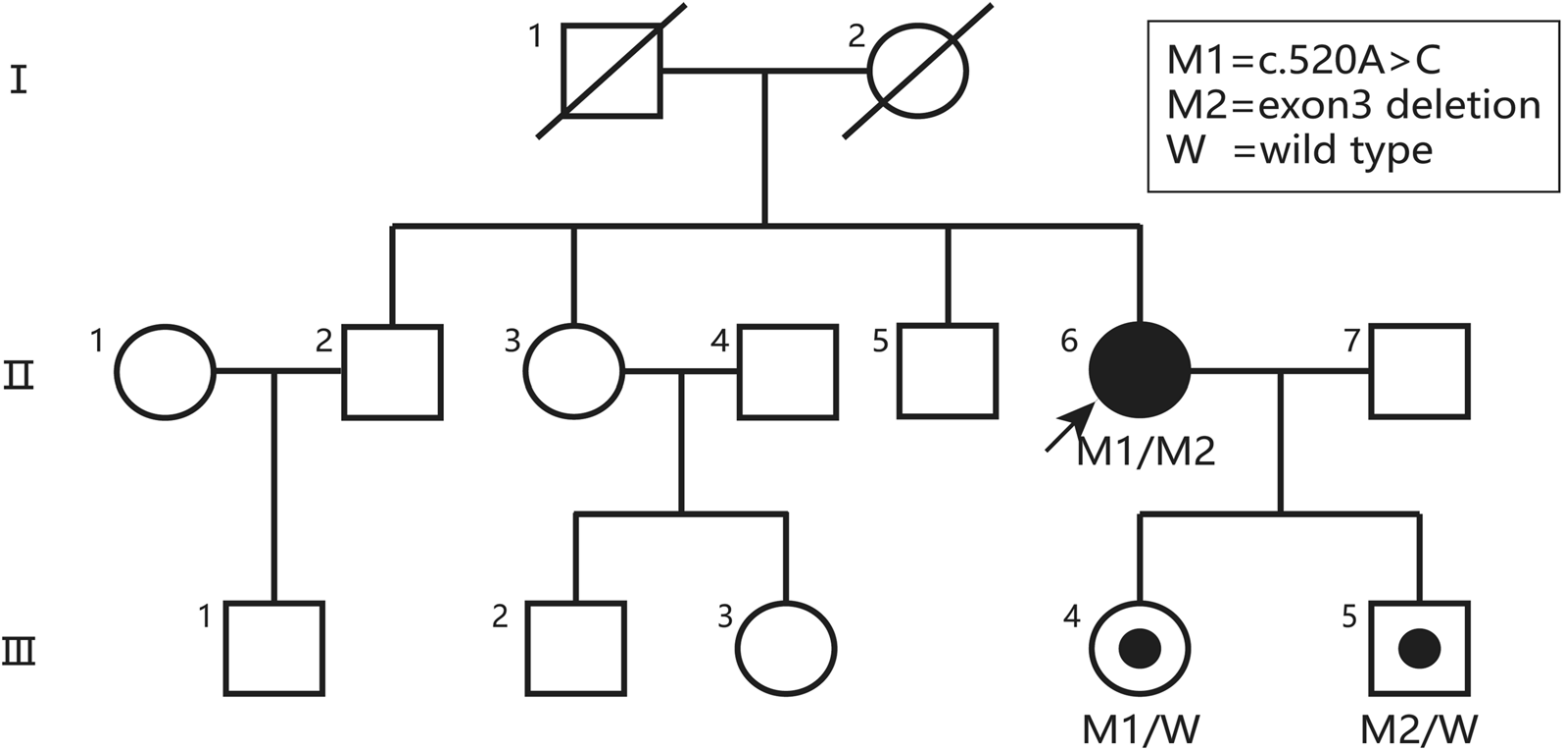
Pedigree of the family including one patient with MCD and two heterozygous recessive carriers. The arrow indicates the proband II-6, the III-4 carries the mutation of c.520A

Except for the proband (Fig. 1II-6) who had eye symptoms, the family members in three generations and deceased relatives had no records of eye diseases (Fig. 1). There was no history of systemic diseases such as mucopolysaccharidosis in this family.

### Clinical features

**Fig. 2:**
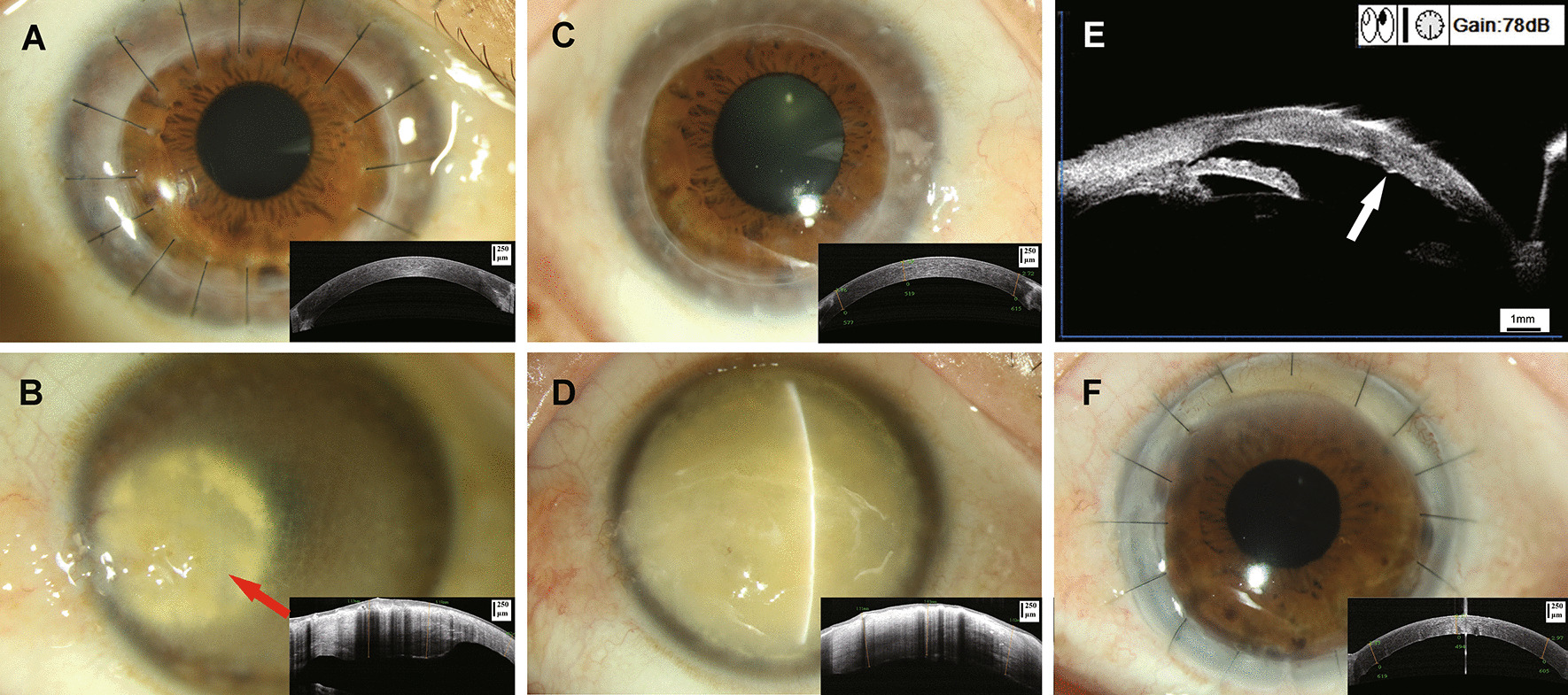
Clinical phenotypes. **A** The right cornea early after PKP was transparent. **B** The left cornea was opaque and edematous combined with stromal inflammation on the nasal side in 2012. **C** The right cornea was transparent, and there was no obvious recurrence at the edge of the graft at 7 years after PKP. The thickness of the graft had no significant difference from that early after surgery. **D** The cornea of the left eye was completely opacque before PKP and was thickened irregularly. **E** Ultrasound biomicroscopy of the left eye before surgery showed irregular thickening of the cornea and guttate excrescences on the posterior elastic layer. **F** The left cornea was transparent after PKP in 2019

The proband presented with vision of 20/800 in the right eye and FC/20 cm in the left eye on her first visit to our hospital. We observed patchy corneal opacity in both eyes and corneal stromal inflammation in the nasal side of the cornea in the left eye with vascular membrane expansion in the cornea (Fig. 2A, B). The dense opacity in the left eye was different from the common MCD. The right cornea recovered transparency with best corrected visual acuity (BCVA) of 20/200 at six months after PKP (Fig. 2A). The proband came to our hospital in 2019 due to poor vision in her left eye. In the right eye, BCVA was improved to 20/25, and the centre and edge of the graft were transparent with no recurrence (Fig. 2C). The endothelial cell count of the graft was 617 cells/mm^2^. We found opacity and thickening in the left cornea (Fig. 2D). Real time OCT revealed an uneven corneal epithelium, irregular mass turbidity under the epithelium, and uneven turbidity of the corneal stroma (Fig. 2D). Ultrasound biomicroscopy showed uneven thickening of the Descemet membrane, guttate excrescences, and endothelial folds (Fig. 2E). To improve the vision of the left eye, a PKP was performed. The graft was transparent, and BCVA was 20/80 at one year after surgery (Fig. 2F).

### Corneal pathology

**Fig. 3:**
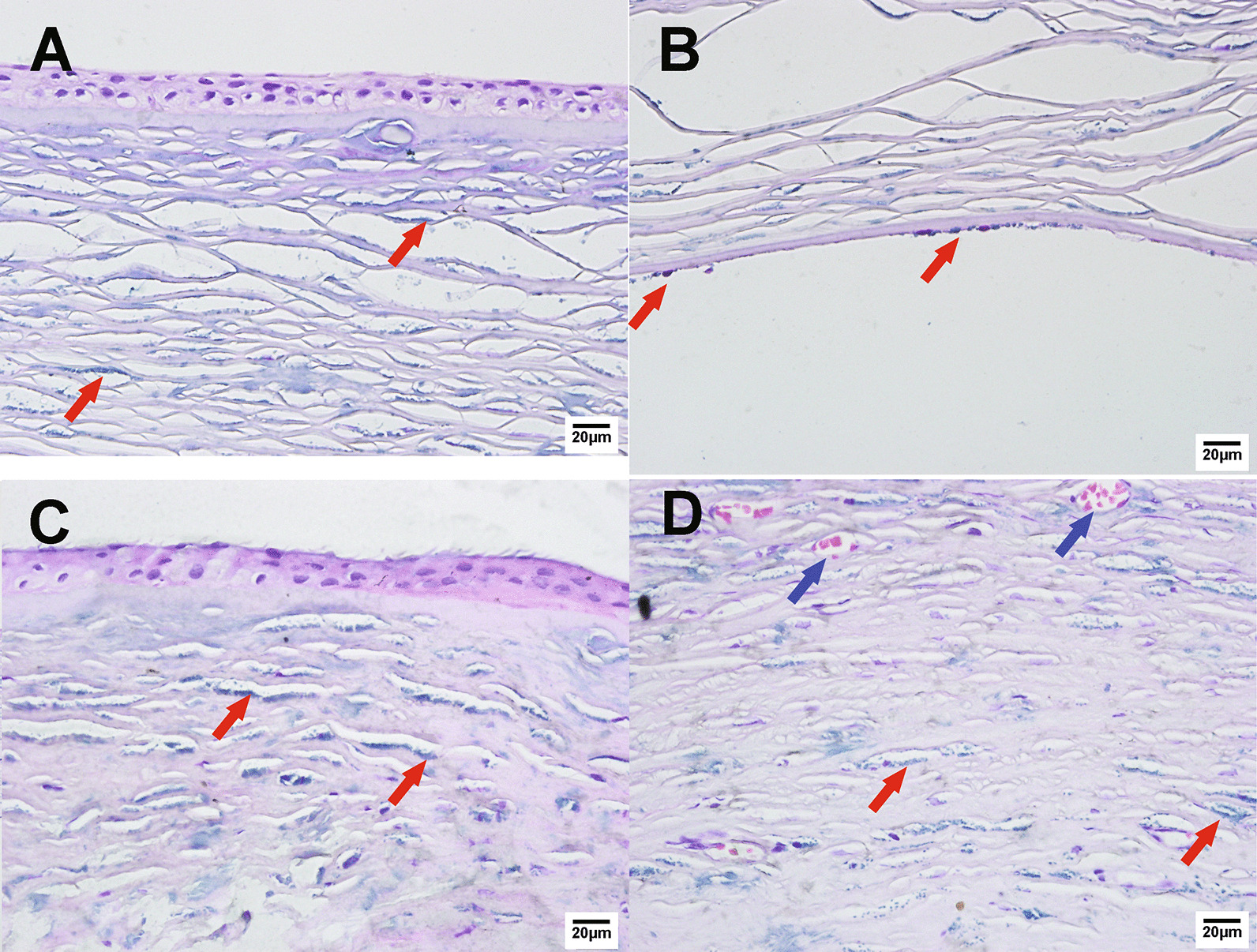
Histopathological staining with colloidal iron. The corneas of the proband were obtained during PKP. **A**, **B** show histopathological staining of the right cornea. Blue plaque deposits were found in the corneal stroma (**A**) and corneal endothelium (**B**). **C**, **D** show histopathological staining of the left cornea. More blue plaque deposits were found in the same layers due to complicated stromal inflammation, stromal thickening, lamellar structure disorder (**C**), and vascular proliferation (**D**). The red arrows indicate the blue plaque deposits, and the blue arrows indicate the neovascularization in the stroma. (400 ×)

Histochemical staining showed that the corneal epithelium was thinning and of uneven thickness. There were a dense deposition of acid mucopolysaccharide in the superficial stroma and a diffuse deposition in the deep stroma (Fig. 3A). Local sub-epithelial mass deposition, positive staining of colloidal iron, and blue particle deposition in the endothelial cells (Fig. 3B) were observed in both eyes. The blue particle deposition in the cornea was denser in the left eye than in the right (Fig. 3C). The cornea of the left eye also exhibited stromal inflammation and eventually developed degeneration with neovascularization (Fig. 3D).

### Gene sequencing

**Fig. 4:**
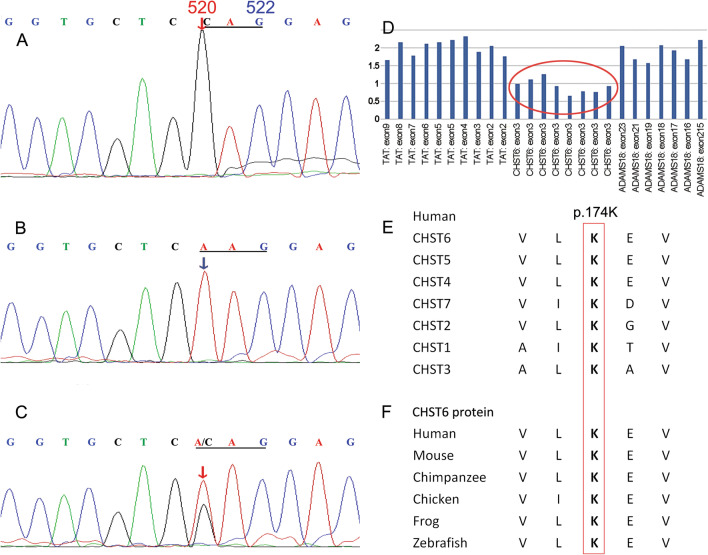
Hemizygous mutations identified by sequencing analysis of the *CHST6* gene. **A** Missense mutation of c.520A>C (p.K174Q) in *CHST6* of the proband. The single peak of c.520C revealed that the c.520A>C mutation of the proband was homozygous or hemizygous. **B** Normal sequences of *CHST6* (Reference Sequence: **NM_021615**) and the *CHST6* sequences of the proband’s son (III-3). The sequense of C.520A revealed that the III-3 was normal or hemizygous carrier. **C** The *CHST6* sequences of the proband’s daughter (III-4). The two peaks of c.520A and c.520C showed the III-4 was a heterozygous carrier. **D** Target exome sequencing revealed a heterozygous deletion of exon 3 of *CHST6* as indicated by the red circle. And the deletion of exon3 was detacted from the proband and the son. The *TAT* gene located on 16q22.2, and the *ADAMTS18* gene located on 16q23.1. The ratio of *CHST6* was obviously lower than the TAT and *ADAMTS18* genes, which showed the deletion of exon 3. **E**, **F** Multiple sequence alignment analysis between human *CHST1-7* protein and other sulfotransferases of animals demonstrated that the novel mutation substituted relatively well-conserved amino acid residues. The red arrows indicate the position of the c.520A>C mutation, and the blue arrow indicates the position of the normal sequence

We identified a novel missense mutation of c.520A>C (p.K174Q) in the exon 3 of the *CHST6* gene (Fig. 4A) as well as exon 3 deletion (Fig. 4D) in the allele of the proband compared with the normal sequence (Fig. 4B). The single peak of c.520C in Sanger sequencing and the low ratio of exon3 revealed that the mutation of c.520A>C and exon 3 deletion constituted a hemizygous variation. A heterozygous mutation of p.K174Q in the *CHST6* gene was detected in the proband’s daughter (Fig. 4C, Fig. 1III-4). The deletion of exon 3 of the *CHST6* gene was detected too in the proband’s son (Fig. 4D). Neither of the two mutations was found in the 1000 Genome, ESP6500, ExAC, and ExAC-EAS population databases. The prediction of SIFT, Ployphen-2, Mutation Taster, and REVEL revealed that they were both deleterious mutations, and GEREP++ predicted the missense mutation in conservative regions. In addition, Multiple sequence alignment analysis between human *CHST1-7* protein and other sulfotransferases of animals demonstrated that the novel mutation substituted relatively well-conserved amino acid residues (Fig. 4E, F).

## Discussion

In this report, we described *CHST6* mutations, including a novel missense mutation of c.520A>C (p.K174Q) and a rare exon 3 deletion mutation, in the members of a family with MCD. Patients with MCD often suffer from vision loss and recurrence of corneal epithelial erosion [1, 7]. In this pedigree, the proband had the onset of white patchy opacity of the corneal stroma and a gradual decrease of visual acuity at 20 years old without obvious epithelial irritation.

MCD can involve the limbus, deep stroma, and Descemet membrane and form irregular nodular turbidity with blurred boundaries. When nodules protrude into the epithelium, they often lead to corneal surface irregularities. When the lesion involves the Descemet membrane, grey appearance and guttate excrescences of the Descemet membrane can be seen. In addition, MCD is usually characterised by a thinner cornea. It has been reported that keratin sulphate in MCD is distributed throughout the cornea [1, 2]. When sulphation is impaired, the corneal opacity often involves the whole cornea. However, the incidence of decompensation is low [7]. In the proband in this study, the opacity expanded to the entire cornea without endothelial decompensation, which is consistent with previous reports [1, 7].

Gene sequence detection is the most important diagnostic criterion for corneal dystrophy and other polygenic diseases [8–14]. In this series, the proband had a missense mutation of c.520A>C (p.K174Q) and an allele exon 3 deletion mutation in the *CHST6* gene. However, the mutations in her daughter, who showed a non-pathogenic p.K174Q heterozygous mutation, and her son, who had a non-pathogenic mutation of the exon 3 deletion, were distinct from the disease in the pedigree. Akama et al. [15] reported that a variant of p.K174R (basic lysine to basic arginine) was a pathogenic mutation. In our study, the basic lysine was replaced by neutral glutamine, which was an important site of enzyme catalysis. Moreover, the multiple sequence alignment analysis between human CHST1-7 protein and other sulfotransferases of animals demonstrated that the upstream and downstream regions of this site were highly conserved (Fig. 4E, F). There have also been reports of pathogenic mutations, such as p.S167F, p.L173F/P, p.V176M, and p.L177GC/G/H [4, 16–19]. The missense mutation of p.K174Q was not detected in the normal population, so we speculate that it may result in abnormal protein function.

The coding sequences of the open reading frames (ORFs) of the *CHST6* gene are within the exon 3, and research has shown that the deletion of ORF could lead to MCD [15, 20, 21]. The deletion of exon 3 can induce dysfunction of the coding gene. In this family, we observed typical clinical features of MCD as well as acid mucopolysaccharide deposits in the cornea of the proband. The *CHST6* mutation conformed to recessive genetic characteristics. Therefore, we speculate that the missing *CHST6* exon 3 and the c.520A>C (p.K174Q) missense mutation contribute to MCD. We could not determine the source of the exon 3 deletion and the c.520A>C (p.K174Q) mutation as the proband’s parents were deceased.

Keratin accounts for more than 65% of total corneal glycosaminoglycans. Synthesised by corneal stromal cells, it exists in all layers of the cornea in a highly sulphated form. When combined with core proteins such as keratocan, keratan sulfate, and other glycosaminoglycans from proteoglycans, it distributes between the main collagen fibres and plays a role in maintaining the transparency of the cornea. Keratan sulfate is soluble in water, but keratin molecules lose solubility when abnormally sulphated and deposited in the extracellular matrix. The deposits can be stained by PAS, Alcian blue, colloidal iron, and metachromatic dyes. Lumican and keratocan, the major proteoglycans of keratin sulfate in the corneal stroma, play important roles in regulating collagen fibril diameters and interfibrillar spacings [22–24]. In addition, the main glycosaminoglycan of the corneal stroma is hyaluronic acid in the early stage of embryonic development. With the development of the cornea, the content of hyaluronic acid gradually decreases while the content of keratin sulfate gradually increases [25–28]. The pathological staining of the cornea of the proband in this study demonstrated the deposition of typical blue-stained glycosaminoglycan particles. Due to the incomplete recording of the early examination results, only the central corneal thickness could be measured through OCT. The corneal slices of the right eye became thin, which is consistent with typical MCD.

However, the left cornea was characterised by corneal stromal inflammation on the nasal side and was obviously thickened and turbid. It differed from the common MCD as the pathology results showed more blue plaque deposits, stromal thickening, lamellar structure disorder, and vascular proliferation. This may be attributed to the infectious factors such as viruses in stromal inflammation and the matrix changes caused by MCD. Nonetheless, there has been no report about MCD secondary to corneal degeneration.

The current research on the pathogenesis of CHST6 mainly focuses on the protein structure of C-GlcNac-6-ST. More than 180 pathogenic or suspected pathogenic mutations including single point mutations in *CHST6* have been reported [5, 7]. However, the phenotype is similar [2]. These genetic mutations may affect the disease through mediating the RNA expression or modifications. Di Iorio et al. [29] found that CHST6 mRNA was in the suprabasal layer of the epithelium, in the stroma and endothelium. The mutations may down-regulate the RNA expression or affect the modifications, which lead to C-GlcNac-6-ST deficiency.

Jin et al. [30] disclosed that as one of the modified molecules on human mRNAs, N4-Acetylcytidine (ac4C) played a key role in the transcriptional translation process and was involved in the occurrence of various diseases, such as infection, inflammation, tumors, and autoimmune diseases. Fei et al. [31] found that amniotic fluid mesenchymal stem cells could repair corneal cold injury in mice by promoting the ETV4/JUN/CCND2 signal axis activation and improving its stability by stimulating ac4C modification of their mRNAs. Thus, corneal epithelial cells of patients with MCD may have low expression of ac4C and insufficient mRNA modification, which cause corneal epithelial damage. To further reveal the pathogenic mechanism of MCD in the molecular expression and regulation levels, it is necessary to detect the changes of the regulation pathway and network in the patient’s tears or aqueous humor [32–34].

Various treatments may be adopted according to the characteristics and progression of MCD. In the early stage of the disease, phototherapeutic keratectomy can be used to improve visual acuity and release eye pain. However, there might be recurrence of MCD within 13.5 months [35]. In the late stage, PKP is an effective procedure to completely remove the corneal opacity. The age of patients with MCD receiving PKP surgery for the first time was reported to be 41 ± 4 years old [36]. Because of corneal transplant rejection and endothelial cell loss after PKP, a second transplantation may be required. A retrospective study of 229 eyes showed that the probabilities of graft survival were 98.1% at 1 year, 89.8% at 5 years, 82.1% at 10 years, and 74.1% at 15 years [37]. Corneal endothelial rejection episodes occurred in 20.0% of grafts. Moreover, deep anterior lamellar keratoplasty (DALK) has been advocated as an alternative treatment. Corneal endothelial density was reduced to 1,000 cells/mm^2^ or less within five years in 21.6% of eyes treated by PKP and in none of the eyes treated by DALK [38]. PKP showed advantages over DALK with a low recurrence rate and better visual acuity postoperatively.

MCD can still recur from 20 months to 30 years after surgery [39], and the average interval before recurrence is 182 months (15.2 years) [36]. The recurrence of MCD often involves the shallow and deep stroma of the outer edge of the implant, which may be related to the recipient corneal cells invading the donor cornea from the periphery to produce abnormal keratan sulphate deposits. The time of recurrence is inversely linked to the size of the implant [37].

In the proband of this pedigree, the centre and margin of the corneal graft were transparent, with a BCVA of 20/25 and without rejection or obvious recurrence seven years after PKP in the right eye. Although the endothelial cell density decreased from 3,036 cells/mm^2^ to 617 cells/mm^2^ in seven years, there was no endothelial decompensation.

## Conclusions

In summary, we discovered a missense mutation of c.520A>C (p.K174Q) in *CHST6* for the first time from a Chinese family with MCD. The left eye of the proband presented stromal inflammation, which differed from typical MCD and interstitial keratitis and may mislead the diagnosis. Moreover, the long-term follow-up outcomes demonstrated that PKP was safe and effective for treatment of MCD.

## Data Availability

The variant has been submitted to the Genome Sequence Archive in National Genomics Data Center, China National Center for Bioinformation, under accession number HRA001367. The raw sequence datasets generated during this study are not publicly available because it is possible that individual privacy could be compromised but they are available from the corresponding author on reasonable request. Public databases used in this study included Human reference genome (GRCH37/hg19) (https://www.ncbi.nlm.nih.gov/assembly/GCF_000001405.13/), 1000 genomes database (http://www.1000genomes.org/), ESP6500 (NHLBI Exome Sequencing Project: https://evs.gs.washington.edu/EVS/), EXAC (The Exome Aggregation Consortium: http://exac.broadinstitute.org/), and EXAC-EAS (EXAC-East Asian: http://exac.broadinstitute.org/).

## Abbreviations

*CHST6*: Carbohydrate Sulfotransferase 6
MCD: Macular corneal dystrophy
PKP: Penetrating keratoplasty
TGFBI: Transforming growth factor beta-induced
BCVA: Best corrected vision acuity
ORF: Open reading frame
DALK: Deep anterior lamellar keratoplasty; SIFT: Sorting Intolerant From Tolerant
PolyPhen-2: Polymorphism Phenotyping v2
REVEL: Rare Exome Variant Ensemble Learner
GERP: Genomic Evolutionary Rate Profiling
